# The effect of methadone and ketamine on quality of recovery in patients undergoing laparoscopic cholecystectomy: a prospective cohort study

**DOI:** 10.1590/1516-3180.2024.0193.R2.18062025

**Published:** 2025-09-19

**Authors:** Leopoldo Muniz da Silva, Ana Clara Mourão Barreto, Rafael Souza Fava Nersessian, Saullo Queiroz Silveira, Helidea de Oliveira Lima, Matheus de Alencar Arraes, Gabriel Silva dos Anjos, Sérgio Martins Pereira

**Affiliations:** I Department of Quality and Patient Safety/Rede D’Or – CMA, São Luiz Hospital/Vila Nova Star/Rede D’Or; Instituto D’Or Pesquisa e Ensino (Idor), São Paulo (SP), Brazil.; II Department of Anesthesia – CMA, São Luiz Hospital/Vila Nova Star/Rede D’Or; Instituto D’Or Pesquisa e Ensino (Idor), São Paulo (SP), Brazil.; III Department of Anesthesia – CMA, São Luiz Hospital/Vila Nova Star/Rede D’Or, São Paulo (SP), Brazil.; IV Department of Anesthesia – CMA, São Luiz Hospital/Vila Nova Star/Rede D’Or, São Paulo (SP), Brazil.; V Department of Quality and Patient Safety/Rede D’Or, Instituto D’Or Pesquisa e Ensino (Idor), São Paulo (SP), Brazil.; VI Department of Anesthesia – CMA, São Luiz Hospital/Vila Nova Star/Rede D’Or, São Paulo (SP), Brazil.; VII Departamento de Anestesiologia – CMA, Hospital São Luiz Hospital/Vila Nova Star/Rede D’Or, São Paulo (SP), Brazil.; VIII Department of Anesthesia, St. Michael’s Hospital, Unity Health; Department of Anesthesiology and Pain Medicine, University of Toronto, Toronto, Canada.

**Keywords:** Methadone, Analgesia, Anesthesia, Methadone, Analgesia, Ketamine, Multimodal analgesia, Anesthesia

## Abstract

**BACKGROUND AND OBJECTIVES:**

Acute pain following laparoscopic cholecystectomy is most intense in the first 24 h. The use of shorter-acting opioids for pain management may contribute to increased postoperative morbidity. The combination of methadone and ketamine has been associated with lower postoperative pain scores and less opioid use. We aimed to determine whether the combination of ketamine and methadone improves the quality of recovery.

**METHODS:**

This prospective cohort study included patients undergoing laparoscopic cholecystectomy. Patients who received either methadone alone or a combination of methadone and ketamine (0.3 mg/kg) were followed up for 24 h after surgery. The primary outcome was the quality of recovery, measured using the quality of recovery-40 (QoR-40) questionnaire. Secondary outcomes included postoperative pain intensity, opioid consumption, and the incidence of nausea and vomiting.

**RESULTS:**

The QoR-40 scores were higher in patients who received methadone and ketamine than in those who received methadone alone [197 (194.7–198) versus 195 (189–197), P = 0.01]. Postoperative pain scores, the incidence of postoperative nausea and vomiting, and postoperative opioid use were similar between the groups. The combination of methadone and ketamine was not associated with lower incidence of moderate-to-severe pain in propensity score analysis.

**CONCLUSIONS:**

Although the combination of methadone and ketamine showed a slight increase in QoR40 scores at 24 h postoperatively, the observed difference between the groups was not clinically significant. Moreover, the absence of a reduction in postoperative pain intensity and similar perioperative opioid consumption between the groups further support the hypothesis that small, isolated doses of ketamine may not be effective in improving recovery quality compared with methadone alone.

## INTRODUCTION

 Acute pain following laparoscopic cholecystectomy is complex in nature and typically most intense on the day of surgery and the subsequent day.^1^ The conventional use of shorter-acting opioids administered in bolus doses for perioperative analgesia may lead to intervals of insufficient pain control due to fluctuating antinociceptive levels.^2^ Conversely, literature indicates that excessive perioperative opioid administration, contrary to its intended purpose, is correlated with increased postoperative morbidity, including pain.^3,4^ Although not fully elucidated, evidence has highlighted the role of N-methyl-D-aspartate (NMDA) receptors in the pathways involved in pain activation.^4^

 NMDA receptor antagonists, such as ketamine and methadone, have been investigated as part of multimodal strategies for managing acute postoperative pain. These agents demonstrate potential for preemptive analgesia by impeding central sensitization to nociceptive stimuli.^4^ Ketamine acts mainly as a competitive antagonist of NMDA receptors, while methadone functions as a long-acting μ-opioid receptor agonist with additional activity at κ- and σ-opioid receptors and also inhibits the reuptake of monoamines and catecholamines.^5^ Brinck et al.^6^ concluded that perioperative ketamine is associated with a reduction on postoperative pain scores and analgesic requirements. Methadone has similarly been proposed as an alternative for perioperative pain management,^7^ including in patients undergoing ambulatory procedures^8^ and surgeries with next-day discharge.^9^

 Although the association between methadone and ketamine has been studied in patients with spinal disorders,^10^ the potential benefits of such a combination in other populations are lacking. Data regarding the effect of this combination on the quality of recovery remain scarce. This prospective cohort study aimed to assess whether adding ketamine to a methadone-based regimen improves postoperative recovery quality in patients undergoing laparoscopic cholecystectomy. The primary outcome was the quality of recovery (QoR-40) score measured 24 h after surgery. Secondary outcomes included postoperative pain at rest and during movement, opioid consumption, and the incidence of postoperative nausea and vomiting (PONV). 

## Materials and Methods

 From January 2022 to July 2022, all patients scheduled for elective laparoscopic cholecystectomy were screened and enrolled after obtaining approval from the Research Ethics Committee (Protocol No. 4.959.204; CAAE: 51393621.4.0000.0087), and written informed consent was obtained. We followed the Strengthening the Reporting of Observational Studies in Epidemiology guidelines.^11^

### Inclusion and exclusion criteria

 Patients were selected based on the daily schedule of elective laparoscopic cholecystectomy. Inclusion criteria included patients aged 18 to 65 years, classified as American Society of Anesthesiologists Physical Status Classification (ASA-PS) I or II, and a body mass index (BMI) of ≤ 30 kg/m ^-2^. Exclusion criteria were as follows: (i) cardiovascular diseases associated with coronary ischemia, (ii) cardiac conduction disorders (defined as QTc > 450 ms), (iii) poorly controlled psychiatric disorders, (iv) significant liver disease (cirrhosis or hepatic failure), (v) preoperative chronic renal insufficiency or failure, (vi) a history of alcohol or drug abuse, (vii) allergy to methadone or ketamine, (viii) acute cholecystitis, (ix) intraoperative cholangiography, (x) chronic opioid use, (xi) concurrent surgical procedures, (xii) unplanned intraoperative endoscopic retrograde cholangiopancreatography, (xiii) bleeding with hemodynamic instability, (xiv) conversion to open surgery, (xv) unplanned transfer to the intensive care unit, (xvi) surgical reintervention, and (xvii) refusal to participate. 

### Perioperative care

 All patients were continuously monitored using electrocardiography, pulse oximetry, non-invasive blood pressure measurement, capnography, a skin temperature probe, train-of-four stimulation, and bispectral index monitoring. Premedication was not routinely administered. Anesthesia was induced with propofol (1-2 mg/kg), remifentanil (1 mcg/kg), and rocuronium (1.2 mg/kg). After induction, all patients received intravenous methadone (0.15 mg/kg) and intravenous dexamethasone (10 mg). Intravenous ketamine (0.3 mg/kg) was administered at the discretion of the attending anesthesiologist. Anesthesia was maintained with remifentanil (0.1-0.5 mcg/kg/min) and targetcontrolled infusion propofol (1-4 mcg/ml) to achieve bispectral index values between 40 and 60 and mean arterial pressures within 20% of baseline measures. At the end of surgery, all patients received intravenous ketoprofen (100 mg), intravenous metamizole (2 g), and intravenous ondansetron (8 mg). Notably, metamizole, a non-narcotic pyrazolone derivative, is the most widely used analgesic in some countries and remains a commonly prescribed drug in Brazil for pain relief.^12^ Intravenous fluids were administered continuously at a rate of 5 ml/kg/h. Hypotension was treated with ephedrine (5 mg), metaraminol (0.5 mg), or fluid boluses. Normothermia was maintained using a convective air-circulation heating system (Bair Hugger; 3M-Switzerland, Rüschlikon, Switzerland). Neuromuscular blockade was reversed with sugammadex, following recommended guidelines. Patients were extubated in the operating room and transferred to the post-anesthesia care unit (PACU). 

 In the PACU, patients experiencing moderate to severe pain (defined as a numeric rating scale [NRS] score > 3) received intravenous morphine: 0.03 mg/kg for moderate pain and 0.05 mg/kg for severe pain. The goal was to reduce pain to an NRS score of 3 or lower (0, no pain; 10, worst pain imaginable). Patients presenting with PONV were treated with 30 mg of intravenous dimenhydrinate. In the ward, all patients followed the institutional protocol and received intravenous metamizole (2 g every 6 h), intravenous ketoprofen (100 mg every 12 h), intravenous ondansetron (4 mg as needed for nausea), and intravenous tramadol (100 mg every 6 h if NRS > 3). Patients who required tramadol were reassessed 30 min after administration. For patients with moderate-to-severe pain after administration of metamizole, ketoprofen, and tramadol, a medical evaluation was requested, and intravenous morphine (2 mg) was administered every 15 min until mild pain was achieved. 

### Data collection and outcomes

 Patients were followed for 24 h, beginning with the preoperative assessment and continuing through the postoperative period. In the preoperative area, a research team member calculated the Apfel score for all patients. Data collected from the anesthesia records included the time to extubation, duration of surgery, and cumulative intraoperative doses of remifentanil and propofol. Extubation time was defined as the interval between the end of anesthetic drug infusion and the patient’s eye opening in response to verbal commands. In the PACU, patients were assessed for pain every 15 min. Pain severity was categorized using the NRS: scores of 1–3 indicated mild pain, 4–6 moderate pain, and 7–10 severe pain. Nurses recorded the total amount of intravenous morphine administered, instances of PONV, and postoperative complications such as cardiac arrhythmias and respiratory depression. Residual sedation was assessed at 15 and 30 min after PACU admission using the modified Wilson sedation scale.^13^

 In the inpatient ward, pain assessments were conducted at rest and during movement within the first hour and again 6 h after admission. Nurses recorded episodes of PONV during the first 24 postoperative hours. Following the institutional protocol, patients were instructed to ambulate with assistance 3 h after ward admission and could request additional pain assessments if needed. All relevant data were recorded, and opioid doses administered in the ward were converted to morphine equivalents. 

 At 24 h post-admission and before hospital discharge, a research team member, blinded to the patient’s group allocation, administered a questionnaire to assess the quality of postoperative recovery. The Quality of Recovery questionnaire is a patient-reported tool that evaluates physiological values, functional recovery, and patient-reported outcomes.^14^

### Statistical analysis

 The sample size was determined from the available data, i.e., all patients who underwent elective laparoscopic cholecystectomy at our institution from January 2022 to July 2022. No *a priori* power calculations were performed. However, a *post hoc* power analysis was performed using the actual sample size and parameter estimates derived from the dataset. For an effect size of 0.5 total QoR-40 score in the methadone–ketamine (MK) group compared with the methadone-only (ME) group [197 (194.7–198) versus 195 (189–197), respectively] and a significance level of 5%, a t-test yielded a critical t-value of 0.66 and statistical power (1-β) of 0.82. 

 Categorical variables are presented as absolute values and percentages. The normality of continuous variables was assessed using the Shapiro–Wilk test, with results reported as mean (SD) and median (IQR). Given the non-normal distribution and clear negative skewness of the original QoR-40 score data, the Wilcoxon rank-sum test was used for nonparametric comparisons at each time point. Internal reliability of the QoR-40 was assessed using Cronbach’s alpha. All outcomes were analyzed using univariate logistic regression. 

 To identify independent predictors of moderate-to-severe postoperative pain, a multivariate logistic regression model was used. Variables with P-values < 0.20 in the univariate analysis were considered candidates, and stepwise selection retained those with P-values < 0.10. Effect sizes were expressed as odds ratios (ORs) with 95% confidence intervals (95% CIs). To adjust for potential confounding due to the non-randomized use of ketamine, inverse probability treatment weighting (IPTW) was applied. This method accounted for imbalances in confounding factors between the ME and MK groups. Propensity scores (PSs) were generated using a wide array of independent variables: age, sex, BMI, diabetes mellitus, hypertension, ASA-PS classification, history of nausea and vomiting, Apfel score, extubation time, duration of surgery, cumulative doses of remifentanil and propofol, Wilson sedation score, NRS > 3 in the PACU, and incidence of PONV in the PACU. Patients in the MK group were weighted by the inverse of the PS, while those in the ME group were weighted by the inverse of (1-PS), thus forming the IPTW-adjusted cohort. Covariate balance before and after IPTW adjustment was assessed using standardized mean differences, with a standardized mean difference < 10% considered optimal and < 20% acceptable.^15^ IPTW-adjusted logistic regression models were used to estimate the odds of moderate-to-severe pain, improving the validity of causal inferences in this non-randomized setting. 

 All P-values were calculated using two-tailed analyses, and a cut-off of < 0.05 was adopted to reject the null hypothesis. R software (version 3.4.4; R Foundation for Statistical Computing, Austria) was used for all analyses. 

## RESULTS

**Figure 1:**
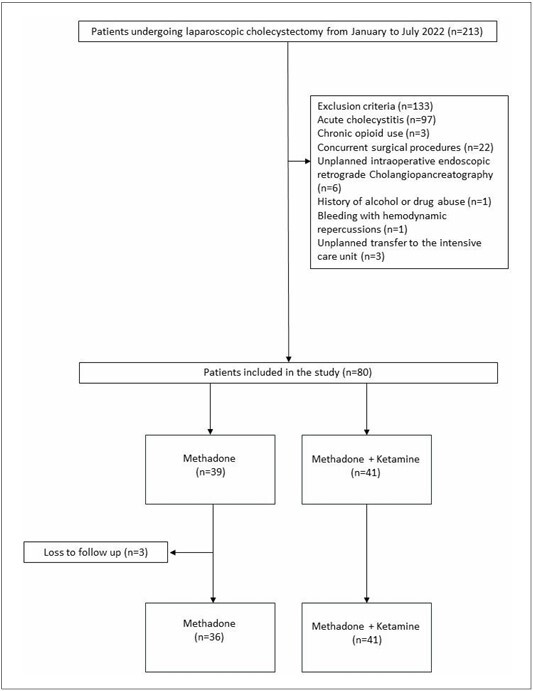
Study flow diagram.

 A total of 213 patients were screened from January 2022 to July 2022. After excluding 133 patients and accounting for three patients lost to follow-up, the final sample consisted of 77 patients (**Figure 1**). Among these, 37 patients received methadone at 0.15 mg/kg, and 41 patients received a combination of methadone 0.15 mg/kg and ketamine 0.3 mg/kg. The mean age of the included patients was 39 ± 9.6 years, and the average BMI was 27.4 ± 2.2 kg/m^2^. Regarding comorbidities, 3% of the patients had type II diabetes, and 14% had hypertension. No statistically significant differences were observed between the ME and MK groups in terms of age, BMI, sex, ASA-PS classification, comorbidities, APFEL score, and smoking habits (**Table 1**). 

**Table 1:** Baseline characteristics, anesthetic management, and postoperative residual sedation in the PACU

		**Overall**		**Groups**				**P value**
				**Methadone (ME)**		**Methadone and ketamine (MK)**		
Age, mean (SD), yr		38.9	(9.6)	38.6	(10.6)	39.2	(8.5)	0.75
BMI, mean (SD), kg/m^2^		27.1	(6.8)	27.4	(5.7)	27.1	(6.1)	0.45
ASA-PS classification								
	I, n (%)	36	(46)	24	(58)	12	(33)	0.05
	II, n (%)	41	(53)	17	(41)	24	(67)	
Diabetes, n (%)		2	(3)	1	(2)	1	(3)	1
Arterial hypertension, n (%)		11	(14)	4	(10)	7	(19)	0.38
History of nausea and vomiting, n (%)		1	(1)	0	(0)	1	(3)	0.95
Smoking, n (%)		8	(10)	6	(15)	2	(6)	0.35
APFEL score								
	1, n (%)	10.4	(8)	4	(10)	4	(11)	0.9
	2, n (%)	48	(37)	19	(46)	18	(50)	
	3, n (%)	41.6	(32)	18	(44)	14	(39)	
Extubation time, mean ± SD, min		8.8	(4.1)	8.1	(4.1)	9.6	(4.1)	0.11
Duration of surgery, mean ± SD, min		55	(45–65)	55	(43–65)	60	(51.5–63.5)	0.31
Cumulative dose of remifentanil, median (IQR), μcg*		545	(409.6–849.6)	520	(409.6–698.4)	687.2	(408.5–954.6)	0.21
Cumulative dose of propofol, mean (SD), mg*		710.2	(274.4)	678.2	(283.5)	746.6	(262.9)	0.28
Wilson sedation score								
	1, n (%)	35	(45)	22	(54)	13	(36)	0.19
	2, n (%)	42	(54)	19	(46)	23	(64)	

Abbreviations: BMI: body mass index; ASA-PS classification: The American Society of Anesthesiologists Physical Status; yr: years

* Total cumulative dose of anesthetics administered during the intraoperative period.

 Details regarding anesthetic management and postoperative residual sedation in the PACU are shown in **Table 1**. Extubation time was not prolonged in the MK group compared with the ME group. Surgical duration did not differ between the study arms. No difference was found in cumulative intraoperative doses of remifentanil [mcg, 520 (409.6−698.5) versus 687.2 (408.5–954.6), P = 0.21] and propofol [mg, 678.2 (283.5) versus 746.6 (262.9), P = 0.28] between the ME and MK groups. Analysis of postoperative sedation using the Wilson sedation scale revealed no difference between the groups. 

 Postoperative sedation levels in the PACU were evaluated using the modified Wilson sedation scale. In the ME group, 54% of patients (n = 20) received a score of 1, indicating that they were alert and oriented, while 46% (n = 17) scored 2, indicating wakefulness with drowsiness. In the MK group, 36% (n = 15) scored 1 and 64% (n = 26) scored 2. No patients in either group exhibited deeper sedation levels (scores ≥ 3), and no episodes of respiratory depression or delayed emergence were observed. Overall, Wilson sedation scale scores did not differ between the groups (P > 0.05). 

### Primary outcome

 The postoperative scores of the five dimensions of the QoR-40 are summarized in **Table 2**. At 24 h after surgery, the total QoR40 score was higher in the MK group than in the ME group [197 (194.7–198) versus 195 (189–197), P = 0.01]. No significant differences were observed between groups in the dimensions of physical independence and psychological support. By contrast, the ME group had significantly lower scores in physical comfort, emotional state, and pain than the MK group (P < 0.05). The overall Cronbach’s alpha for the total QoR-40 score for both groups was 0.93. 

**Table 2:** Primary outcome: Subcomponents of the Quality of Recovery-40 score 24 h after surgery in patients receiving either intraoperative methadone (ME) or methadone ketamine combination (MK)

**Subcomponents of the Quality of Recovery-40 score**	**Groups**				**P value**
	**ME**		**MK**		
Physical comfort (60)	58	(57–59)	59	(57–60)	0.02
Emotion state (45)	45	(42–45)	45	(44–45)	0.04
Physical independence (25)	24	(22–24)	24	(22.75–24)	0.33
Psychological support (35)	35	(34–35)	35	(35–35)	0.13
Pain (35)	34	(33–34)	34	(34–35)	0.01
Total score (200)	195	(189–197)	197	(194.7–198)	0.01

The maximum score for each dimension is reported in parentheses; values are medians (25th and 75th percentiles).

### Secondary outcomes

 Pain levels, postoperative complications, nausea and vomiting, and the accumulated dose of morphine in the PACU and ward are shown in **Table 3**. The percentage of patients reporting an NRS > 3 at rest and during movement did not differ between groups at PACU admission and at 1, 6, and 12 h after admission to the ward. No episodes of respiratory depression or cardiac arrhythmia occurred in the PACU. In addition, no significant differences were found in the incidence of PONV between the two groups. The total dose of morphine administered in the PACU (in milligrams) did not significantly differ between the groups [5.4 (2.6) vs. 6.8 (3.2), P = 0.48]. Similarly, in the ward, no significant differences were observed in the dose of morphine equivalents administered [ME 4.0 (5.6) versus MK 4.8 (5.7), P = 0.28] or in the incidence of PONV. Within the first 24 h postoperatively, the incidence of moderate-to-severe pain in the ward showed no difference between the ME and MK groups. The total morphine dose administered during this period also did not differ between groups (P = 0.28). 

**Table 3:** Secondary outcomes: Pain at rest, pain with movement, cardiac arrhythmias, respiratory depression, nausea and vomiting, and total dose of morphine

		**Overall**		**Methadone**		**Groups**		**P value**
						**Methadone and ketamine**		
Level of pain at rest (NRS > 3)								
	*On PACU admission, n (%)*	43	(56)	24	(58)	19	(53)	0.78
	*1 h after admission to the ward, n (%)*	15	(19)	7	(17)	8	(22)	0.78
	*6 h after admission to the ward, n (%)*	5	(6)	3	(7)	2	(6)	1
	*12 h after admission to the ward, n (%)*	4	(5)	2	(5)	2	(6)	1
Level of pain with movement (NRS > 3)								
	*1 h after admission to the ward, n (%)*	3	(4)	2	(5)	1	(3)	1
	*6 h after admission to the ward, n (%)*	5	(6)	3	(7)	2	(6)	1
	*12 h after admission to the ward, n (%)*	4	(5)	2	(5)	2	(6)	1
PACU								
	*Cardiac arrhythmias, n (%)*	0	(0)	0	()	0	(0)	1
	*Respiratory depression, n (%)*	0	(0)	0	()	0	(0)	1
	*Nausea and vomiting, n (%)*	5	(6)	3	(7)	2	(6)	0.98
	*Total dose of morphine, mean (SD)*	5.9	(2.2)	5.4	(2.6)	6.8	(3.2)	0.48
Ward								
	*Total dose of morphine equivalents, mean (SD), mg*	4.3	(5.2)	4.0	(5.6)	4.8	(5.7)	0.28
	*Nausea and vomiting, n (%)*	11	(14)	6	(14)	5	(14)	0.95

Abbreviation: NRS: numeric rating scale.

 Univariate analysis revealed no factors independently associated with moderate-to-severe postoperative pain in the PACU. In the multivariate analysis, adjusted using PS methods, the combined administration of methadone and ketamine was not significantly associated with the occurrence of moderate-to-severe pain compared with methadone alone (OR = 0.79; 95% CI; 0.292.12). However, longer surgical duration was slightly associated with a higher incidence of postoperative pain (OR = 1.02; 95% CI; 1.004–1.05) (**Table 4**). 

**Table 4:** Factors associated with the incidence of pain (NRS > 3) at the post anesthesia care unit—summary of propensity-weighted analysis

		**Propensity-weighted analysis**		
		**Estimate (OR)**	**95% CI**	**P value**
Groups				
	*Methadone*	1		0.64
	*Methadone and ketamine*	0.79	0.29 – 2.12	
Duration of surgery, min		1.02	1.004 – 1.05	0.03
Cumulative dose of propofol, mg		1	0.99 – 1.001	0.45
Smoking		7.5	1.1 – 149.4	0.07

These estimates correspond to the prevalence ratio (OR). CI = confidence interval; propensity score was estimated using a logistic regression model.

## DISCUSSION

 This prospective cohort study aimed to evaluate whether adding ketamine to a methadone-based regimen would influence QoR-40 scores in young patients undergoing laparoscopic cholecystectomy. The results demonstrated a slight improvement in QoR-40 scores 24 h post-surgery in patients who received both methadone and ketamine compared with those who received methadone alone. However, incidences of moderate to severe pain and PONV showed no differences between groups, as was opioid consumption. These findings suggest that administering low-dose ketamine at anesthesia induction did not substantially impact pain management or postoperative complications in this cohort. No episodes of cardiac arrhythmia or respiratory depression were reported. 

 Postoperative recovery is a key outcome for anesthesiologists, as it directly influences patient satisfaction.^16^ The QoR-40, a validated 40-item questionnaire, is regarded as one of the most effective tools for assessing the complex and multidimensional aspects of recovery following general anesthesia and surgery.^16^ Nonetheless, interpreting QoR-40 scores requires caution. Both groups in this study presented high QoR-40 scores, indicating good recovery^17^ and an acceptable symptom burden.^18^ Although statistically significant differences were observed in individual domains such as pain, comfort, and emotional well-being, the overall difference in QoR-40 scores between groups did not reach the 6.3-point threshold, which is the proposed cutoff for clinical significance.^18^ While QoR-40 scores are applicable to assess postoperative patient recovery following any type of procedure, lower scores might be expected in patients undergoing more complex and painful surgeries. In such cases, an intervention such as the one we proposed could potentially offer greater benefit. Conversely, young and otherwise healthy patients undergoing laparoscopic cholecystectomy report higher QoR-40 scores, which may reduce the observable effect size of additional interventions. Within this context, the addition of ketamine to a methadone-based regimen did not result in a clinically meaningful improvement in postoperative recovery. 

 Multiple factors may contribute to the lack of differences observed not only in QoR-40 scores, but also in postoperative pain scores and opioid consumption between the groups. A key consideration is the timing of administration, whether ketamine was given before or after skin incision, which may play a critical role.^19,20^ Ketamine is a "use-dependent" drug: it blocks NMDA channels only after they have been activated by intense or repeated noxious stimuli.^21^ In other words, the more severe the pain, the more efficient ketamine tends to be. Therefore, the additive analgesic effect of ketamine would be expected to be more pronounced in surgeries associated with higher pain potential. A retrospective cohort study^22^ involving 115,775 patients from 105 different institutions examined postoperative pain in 179 procedures. Despite the complex nature of laparoscopic cholecystectomy, the median NRS was 5, ranking it 94th in plain intensity. Conversely, three of the six surgeries with the highest pain scores were major spinal surgeries. These variations in pain stimuli among different surgical types may explain why low-dose ketamine has demonstrated efficacy as an adjuvant in highly painful orthopedic procedures^23^ but not in laparoscopic cholecystectomy, as observed in our findings. 

 Another key component that may affect ketamine’s effectiveness is the administration regimen.^24^ Continuous intravenous infusion throughout surgery and into the recovery phase is generally more effective for preventing post-surgical pain; however, this approach seems impractical for short-duration surgeries.^25^ In an animal model, subanesthetic doses of ketamine greatly alleviated provoked pain by preventing hyperalgesia and the development of opioid tolerance.^26^ Nonetheless, in patients undergoing laparoscopic cholecystectomy, different ketamine doses administered before skin incision failed to produce differences in either QoR40 scores or postoperative pain scores compared with placebo.^19^ Similarly, the PODCAST trial,^27^ where patients were given placebo, 0.5 mg/kg or 1 mg/kg of ketamine, did not show a reduction in postoperative opioid consumption or pain scores. The lack of benefit from a single dose may be attributed to ketamine’s rapid decline in plasma concentration.^24^ For instance, following a bolus dose, plasma concentrations fall below 150 ng/ml within 10 min for a 0.5 mg/kg dose and within < 25 min for a 1 mg/kg dose.^21^ Hence, the dose and method of administration (i.e., bolus instead of a continuous infusion) in our study might have been insufficient to reach steady plasma concentrations necessary to reduce moderate to intense postoperative pain in patients receiving ketamine. 

 All the patients received intravenous methadone, ketoprofen, and metamizole. Multimodal analgesia has been associated with reduced opioid consumption and improved postoperative pain scores, but not with improved quality of recovery.^28^ Other adjuvants, such as intravenous acetaminophen, which has been demonstrated to be more effective than ketamine in reducing postoperative pain,^29^ are not available in Brazil and therefore was not administered. Concurrently, no increase in adverse effects commonly associated with ketamine, such as nausea and vomiting, was observed. Although higher doses of ketamine might offer more robust pre-emptive analgesia, such doses could also increase the occurrence of emergence hallucinations, nightmares, and other side effects that may limit its use, particularly in short-duration surgeries.^23^

 Although methadone use in the operating room has increased, the literature presents conflicting findings regarding its perioperative administration and its association with ketamine. Compared with shorter-acting opioids, one study found no association between methadone and lower pain scores or improved QoR-40 scores.^30^ By contrast, Kharasch et al.^9^ showed that a single dose of methadone reduced opioid requirements in next-day discharge patients. Administration of methadone in that context may have decreased the effect size between the groups. The potential benefits of methadone and ketamine remain unclear. An experimental rat model of neuropathy suggested a supra-additive effect between ketamine and methadone,^31^ a phenomenon observed in patients undergoing spine surgery,^32^ where the combination led to a greater-than-expected reduction in opioid use and postoperative pain scores.^33^

 Despite the potential analgesic benefits of the MK combination in short-duration surgeries such as cholecystectomy and the use of low doses of ketamine after induction, our study did not find synergistic analgesic effects. A recent cohort study in cardiac surgery reported that even with higher doses of both methadone and ketamine (approximately twice those used in our study), the observed benefit was limited to delaying the time to first rescue opioid administration, without a lasting effect beyond the first postoperative day.^34^ These findings suggest that the analgesic synergy between ketamine and methadone may be highly context-dependent and influenced by surgical invasiveness, nociceptive load, duration of noxious stimuli, and the timing and method of ketamine delivery. 

 This study has some limitations. First, the absence of randomization and the allocation of patients at the discretion of the attending anesthesiologist introduce a significant risk of selection bias. However, **Table 1** shows that the groups were similar at baseline. Second, although laparoscopic cholecystectomy presents a risk for long-term pain management, with chronic pain occurring in up to 10–40% of cases,^35,36^ and perioperative methadone has been associated with lower pain scores at 3 months after surgery,^37^ our follow-up was specifically designed for the early postoperative period and did not evaluate long-term effects. Third, the use of single-dose ketamine at the beginning of surgery may have been insufficient to reduce the incidence of moderate-to-severe pain upon PACU admission. Finally, neither methadone nor the MK combination provided sufficient analgesia in the PACU for most patients, suggesting a need for further studies comparing different doses of intravenous methadone alone^9^ or in combination with ketamine. At 1 and 6 h after ward admission, 81% and 93% of patients, respectively, reported NRS scores < 3. 

 We conclude that small doses of ketamine, when administered as part of a multimodal analgesic regimen, do not improve the quality of recovery, as assessed using the QoR-40 questionnaire, following methadone-based anesthesia for laparoscopic cholecystectomy. Although the MK combination showed a slight increase in the quality of recovery scores 24 h postoperatively, the observed difference between the groups was not clinically significant. Further research is warranted to evaluate the impact of different pain management approaches, considering both short- and long-term effects of MK combination on postoperative recovery. 
